# Letter from Paris

**Published:** 1890-08

**Authors:** H. E. Hayd

**Affiliations:** Paris


### Correspondence.

### LETTER FROM PARIS.

*The Apostoli Clinic—Electro-therapy applied to the Treatment of Diseases of Women—A Buffalo Doctor's Opinion of the famous Electrologist and his Work—He is pleased—The "Yarns" heard in America are all Bosh as to the Suffering the Treatment causes—Apostoli is enthusiastically defended.*

It might interest the readers of the Buffalo Medical Journal to hear what is being done in the hospitals of Paris.

First of all, I may say that Paris is not a good place to do post-graduate work. The hospitals are widely separated from one another, and the hours of service are in the morning. Moreover, there are but few special hospitals; in fact, the abdominal and gynecological surgery is done by the general surgeons connected with the various large institutions.

Fortunately for the medical man who is visiting Paris and anxious to do some good general work, the Rue du Jour, or Apostoli clinic, takes place in the afternoons of Tuesday, Thursday, and Saturday. So, in this respect, it does not interfere with his work in the general hospitals. Like other dispensaries and private clinics, it is decidedly more attractive inside than out. In fact, one's first impression of this somewhat noted institution is an experience similar to Lawson Tait's. At first one shrinks back, and wonders whether women are compelled to walk through that gloomy court and ascend two staircases to get their medical treatment. The hospital consists of five rooms, plainly furnished, but very clean and nicely cared for, with an average attendance of from thirty to thirty-five patients daily.

Besides Dr. Apostoli, there are two assistants, and one of these, his *chef*, treats the patients. The histories of the cases are taken very carefully, and daily records kept. When a patient is seen for the first time, the history is read, an examination made, and the course of treatment outlined and begun at the next visit. The diagnosis is made, and several medical men (and often one sees visitors of national reputation present) are invited to corroborate it, if possible, and give reasons for their opinions. I must say I have been particularly pleased with Apostoli, and can, in all fairness, say that he is a brilliant diagnostician, and what work he does is done with an earnestness and honesty which are truly commendable. What his earlier utterances and claims may have been, I know not, but the position which he now takes in reference to electro-therapy, as applied to diseases of women, is not as extravagant as we in America have supposed. He is a man of great individuality and force of character, and thoroughly sincere in his work. His attention to strangers, his courtesy and politeness, and his desire to have the details of treatment thoroughly understood by them, is very gratifying indeed. Like other men who have worked in special lines, he has formed opinions which he, by his argument and experience, is prepared to substantiate. Much that he says I cannot say that we all agree with him in, yet I am often struck with the probable truth of his utterances. He believes but little in pessaries and supports of any kind, and but very exceptionally employs them. Moreover, he takes the position that *most* lacerated cervices and misplaced wombs are the cause of little trouble in themselves, and only give rise to symptoms when some periuterine inflammation is also present.

One sees a great many fibroids of all sizes and in various locations. Some are extremely large and have been under observation for many years. There is no doubt that the distressing symptoms that these women complained of have been relieved, and they have been enabled to work with comfort and satisfaction. By careful measurement with the tape externally, from various prominent points, and with a small instrument which is used to measure the thickness of the skin and superficial fat in the abdominal walls, one can easily see that, in some cases, a diminution in the size of the tumor has taken place; and, in exceptional cases, the tumor has disappeared. I sometimes feel that upon the grounds of cosmetic surgery, even although no active symptoms are present, some of these very large tumors ought to be removed, and particularly when seen in young women. I have visited the clinic regularly, and can say that the work done is good. The strictest antiseptic precautions are observed. All patients have an injection before and after a treatment, and all electrodes are placed in boiling water, and afterward in a saturated solution of iodoform.

I have been most interested in the use of the faradic current, and in galvano-puncture. I have seen many women extremely sensitive to pain — indeed in whom the lightest pressure over the womb and ovaries caused agonizing suffering — relieved of all their pain after a fifteen minutes' seance with the faradic current of tension.

In exceptional cases one or two treatments has cured the subjective evidences of their trouble, while upon examination the pathological condition was the same as upon the first application, showing us that what Apostoli claims is evident, viz: that, although the gross manifestations of the disease are not overcome, the symptoms are relieved and the flow permanently so.

I have also seen much good from galvano-puncture. After having treated a woman suffering with a mass of cellulitis for six weeks with positive galvanism — which by the way is nearly always employed, as most patients suffer from pain or hemorrhage — with little benefit, Dr. Apostoli punctured the spot with his usual precautions, with much satisfaction to himself and gratification to his patient. The punctures are never deeper than one-eighth of an inch, and the needle employed has a small point, protected by a sheath up to the spot desired to enter the tissues. The strength of current is twenty-five to thirty ma. positive galvanism. Resolution took place and no bad symptoms developed. The yarns, that we have heard in America of the agony these women are made to suffer is all bosh, because one of the first rules of all electro-therapy is to stop the current if not well borne by the patients. Some patients take with ease, and without flinching, 130 to 150 ma. of current. Much care is taken to see that the skin is in no place abraided, and also that the clay-pad is thick over all sensitive areas.

At present there is quite a revolution of feeling in Paris among the profession, as to the claims of Apostoli. The operating surgeons say electricity is no good, and will do nothing for the diseases said to be benefited and cured by Apostoli and his followers. At a meeting of the Society of Practical Medicine of Paris, of which Apostoli is a member, a commission was appointed to investigate his work at his request. Consequently, every day a number of old patients treated years ago are examined by one of this commission, and their present condition noted and compared with that previous to treatment. The new patients are carefully examined, and a diagnosis made and compared with that of Apostoli, and the treatment is begun. This naturally makes the work very interesting to us all, and each one of us visiting the clinic are delighted to see how frank and honest Apostoli is in all of his work. To say that he is unscientific and uneducated is unfair, and to accuse him of quackery and dishonesty is an infernal libel. Enthusiastic? yes; one capable of working with indefatigable energy, true; and, at the same time, full of a desire to do what is best for those women who place themselves under his care. In this impression I am sure I am borne out by every man who is to-day visiting the Rue du Jour.

I have attended Terrier and Champonnier, and other solid abdominal surgeons in Paris, and I believe the best work is done by Terrier. His hospital — Hôpital Bechat — is a modern institution, and thoroughly equipped for all kinds of scientific work. Here asepsis is arrived at. He has a splendid operating room, with glazed walls and ceilings, and the floor of cement. It is divided into two portions, separated by iron doors. The one room is for old suppurative cases, and the other for non-septic ones. The instruments are sterilized by heat, and put into sterilized hot water. The napkins and towels are also treated by a special process, and made sterile. No antiseptics are used, excepting to wash the body of the patient and the operator's hands, and salve or iodoform is applied on the wound, with antiseptic gauze over it.

I saw him perform an abdominal hysterectomy last week, for a very large uterine fibroid. An incision was made about two inches above the umbilicus, extending to the pubes. There were no adhesions; consequently, when the peritoneal cavity was fully opened, the great mass could be rolled out of the abdomen. The interesting feature was the treatment of the pedicle. A long steel bodkin was pushed through the mass as near the vaginal vault as possible. A piece of rubber tubing — solid — was firmly drawn and fully stretched under this steel pin, and tied with silk. The tumor was amputated, and the pedicle was left about an inch in length. The center was dissected out and seared over with the thermo-cautery, then the edges were brought together like the flaps of a stump and sewed firmly and finely with silk sutures. The steel wire was removed and the stump was returned into the abdominal cavity and the rubber ligature left in situ. (?) It was a very brilliant operation, and made one feel proud of the triumphs of surgery. I have also seen him perform many other tube and ovary operations, and a few days ago a hysteropexie or ventro-fixation, which also pleased me very much. The woman was fifty years of age, and suffered from extreme procedentia uteri, complicated with pyosalpingitis. An abdominal incision was made of good length — in fact, these men always make large openings — and the tubes and ovaries carefully removed. Both tubes (?) were cystic and filled with pus. The abscess was then pulled up and four silk sutures were passed through its anterior surface, leaving about one inch of uterine tissue within their grasp. Each suture was carried into the peritoneum of the corresponding side, and then firmly drawn together; holding, therefore, in their grasp, the uterine wall and the peritoneum of the incision. The rest of the peritoneum was picked up and sewed, then the fascia, and finally the skin. A drainage-tube was left in the abdominal cavity.

Dr. Terrier told me he knows of a woman in whom a hysteropexie was performed — the tubes and ovaries being left in — who conceived and carried her ovum to term. Unfortunately one does not see only brilliant, good, and justifiable surgery; but, on the contrary, the most wholesale mutilation of women. It is only a few days ago when one of the first men in Paris diagnosed an enlarged and painful ovary, on the right side. Immediately the young woman was placed on the table and an abdominal section made. When the abdomen was opened, the ovary in question was found to be normal, but the opposite tube was slightly bound down; so, in order to take advantage of the incision, this tube and ovary were gouged out. Another similar case was the removal of the ovaries for a small bleeding fibroid, the operator having no faith in other so-called *unscientific procedures.* If Apostoli has done nothing more than demonstrate the possibility of relieving the pain and hemorrhage in these cases, surgery should welcome his work, in the interests of humanity.

And now let me say a word about hemorrhage. I believe we have made mistakes in our technique, when we don't succeed in controlling the bleeding. I have seen cases come to the clinic who bled profusely after an examination. Indeed, I am sure one woman lost at least six ounces of blood. In these cases Apostoli uses the largest carbon electrode that is possible to be introduced into the uterine cavity, and endeavors to touch the whole internal surface of the womb. This treatment lasted about ten minutes, with the effect of completely arresting all hemorrhage for several days. About sixty ma. positive galvanism was given.

I have also attended Guijon at the Hôpital Neckar, but I am not pleased with the French methods of operating for stricture of the urethra. Seldom do they perform a primary urethrotomy, and are satisfied with the use of much smaller sounds than we in America; in fact, the French urethral surgeon practically disregards Otis's ideas of the normal caliber of the urethra.

I shall endeavor to send you another letter before I leave Paris for Berlin.

Dr. Park is in Paris. I saw his name registered at the Banking office.

H. E. HAYD.

PARIS, June 24, 1890.

